# Editorial: Repurposing β-blockers for non-cardiovascular diseases

**DOI:** 10.3389/fphar.2024.1372317

**Published:** 2024-02-09

**Authors:** Ayaz Shahid, Jeffrey Wang, Bradley T. Andresen, S. R. Wayne Chen, Ying Huang

**Affiliations:** ^1^ Department of Biotechnology and Pharmaceutical Sciences, College of Pharmacy, Western University of Health Sciences, Pomona, CA, United States; ^2^ Department of Biochemistry and Molecular Biology, University of Calgary, Calgary, AB, Canada

**Keywords:** B-blockers, cancer, non-cardiovascular diseases, β-adrenergic receptor, sepsis

β-Blockers are a class of drugs that have been approved by the FDA for the treatment of cardiovascular diseases. However, Some β-blockers have been found to be effective in treating other disorders beyond the cardiovascular system, such as CNS conditions, diabetes, cancer, and organ toxicities (Alaskar et al., 2023; Beaman et al., 2023; Chen et al., 2023; Shahid et al., 2023). Furthermore, β-blockers are proposed to act as immunomodulators due to the role of β-adrenergic receptors in immunity (Fjaestad et al., 2022). Regarding the protection against organ toxicity, non-selective β-blockers, such as carvedilol and propranolol, have been found to protect against renal toxicity (Rodrigues et al., 2010; Rezayat et al., 2017). The effects of β-blockers on diseases outside of the cardiovascular system may not necessarily be related to their β-blocking activity. This issue compiles preclinical and clinical studies, along with a review article, on the use of β-blockers for non-cardiovascular diseases. These studies provide evidence for repurposing these FDA-approved drugs for other diseases and identifying new mechanisms for their use beyond β-adrenergic receptor blockade.

According to Massalee and Coa, β-blockers could be a possible treatment for cancer by blocking β-adrenergic receptor signaling, which is associated with tumor growth and immune system suppression. β-Blockers may also work well in combination with chemotherapy by enhancing anti-proliferative, antimitotic, and antimitochondrial properties, leading to better control of tumors and improved therapy outcomes. β-Blockers could also improve cancer immunotherapy by blocking immunosuppressive signaling and boosting the functionality of immune cells such as CD8^+^ T cells. Non-selective β-blockers, which inhibit both β1-and β2-adrenergic receptors, may be more effective in decreasing tumor proliferation and improving overall survival compared to selective β-blockers. More preclinical and clinical studies are needed to confirm the synergistic potential of combining β-blockers with conventional cancer therapies and/or immunotherapies. There are still challenges in understanding the mechanisms underlying the non-cardiovascular effects of β-blockers and how to use these drugs to improve clinical outcomes in non-cardiovascular diseases.

Yang et al. aimed to evaluate the potential association between β-blockers and reduced mortality in patients with sepsis. The study involved analyzing data from two large ICU databases comprising 61,751 sepsis patients, out of which 43.8% received β-blockers. The data set included both selective and non-selective β-blockers administered by intravenous or oral routes. The study found that β-blocker usage was linked to significantly lower in-hospital mortality compared to non-users. Additionally, β-blocker usage was linked to shorter hospital/ICU stays and increased ventilator/vasopressor-free days. The study also found consistent reductions in mortality with β-blocker usage across various patient groups. Although the mechanisms responsible for the association between β-blockers and reduced mortality are still unclear, it is speculated that this effect may be due to reduced sympathetic activation, improved hemodynamics, and controlled inflammation. This study’s limitations include its nature of retrospective design and lack of data on infection sites and inflammatory markers. Overall, this large retrospective study found that β-blocker usage was associated with substantial reductions in mortality and other improved outcomes in sepsis patients. Large scale prospective studies are needed to verify these findings.

Le Bozec et al. examined the relationship between β-blockers exposure and survival outcomes in 182 patients with advanced pancreatic ductal adenocarcinoma (PDAC). Out of the 182 patients, 41 (22.5%) were exposed to β-blockers. The study found that there was no significant difference in overall survival (OS) or progression-free survival (PFS) between patients who used β-blockers compared to those who did not (HR 1.38, 95% CI 0.80–2.39, *p* = 0.25 for OS; HR 0.95, 95% CI 0.48–1.88, *p* = 0.88 for PFS). Similar results were obtained with the use of propensity score methods. Additionally, the use of selective β1-adrenergic receptor blockers was associated with a significant decrease in OS (HR 1.80, 95% CI 1.16–2.80, *p* < 0.01) compared to non-selective β-blocker use. The study suggests that β-blocker exposure is not associated with improved survival outcomes in patients with PDAC. This finding needs further validation through larger prospective interventional studies. The study also highlights some methodological limitations of retrospective analyses of β-blocker use and cancer survival, including time-dependent bias and proper accounting for cardiovascular disease. Overall, in this study there was no association between β-blocker exposure and no improved survival outcomes in PDAC.

Guo et al. used a two-sample Mendelian randomization approach to examine the impact of non-canonical antihypertensive drug targets on oral cancer outcomes. KCNH2 was inhibited by certain β-blockers but not all (Karle et al., 2001; Kawakami et al., 2006). Guo et al. found a link between KCNH2 expression and oral cancer, suggesting β-blocker use may be effective. However, the study has some limitations, including a lack of information of all the drugs taken by the patients and co-occurring diseases of the patients in the populations examined, and a population restricted to Europeans. Furthermore, more evidence is required which β-blockers inhibit KCNH2 at physiological drug concentrations *in vivo*. Thus, this innovative study proposes a potential protective causal association between β-blocker use and oral cancer risk using Mendelian randomization methods.

In conclusion, these studies have indicated that β-blockers may decrease mortality rates and enhance outcomes for patients suffering from sepsis and cancer. Nevertheless, additional research is required to comprehend the impacts of individual β-blockers in these conditions, as different β-blockers exhibit diverse pharmacological and pharmacokinetic properties (Talbert, 2004; Hocht et al., 2014). Although these studies do not provide sufficient evidence to support the use of β-blockers as anti-sepsis or anti-cancer therapy, existing drugs such as β-blockers may be used to prevent or treat cancer, to benefit patients who suffer from diseases that are outside of the cardiovascular system.
